# Two new terrestrial narrow-toed frogs (*Niceforonia:* Strabomantidae, Anura) from remote unexplored mountains in the Ecuadorian Andes

**DOI:** 10.7717/peerj.21182

**Published:** 2026-05-01

**Authors:** Juan P. Reyes-Puig, Mario H. Yánez-Muñoz, Miguel Urgilés-Merchán, Zane Libke, Santiago R. Ron, Julio C. Carrión-Olmedo

**Affiliations:** 1 Fundación EcoMinga, Red de Protección Amenazados, Quito, Ecuador; 2 Unidad de Investigación, Instituto Nacional de Biodiversidad, Quito, Ecuador; 3 Maestría en Conservación y Manejo de Recursos, Pontificia Universidad Católica del Ecuador, Quito, Ecuador; 4 Fundación Oscar Efrén Reyes, Baños, Tungurahua, Ecuador; 5 Reserva: The Youth Land Trust, Washington, Washington, United States; 6 Museo de Zoología, Pontificia Universidad Católica del Ecuador, Quito, Ecuador; 7 Smithsonian’s National Zoo and Conservation Biology Institute, Center for Species Survival, Front Royal, Virginia, United States

**Keywords:** Amphibia, New narrow toed frogs, Niceforonia, Tropical Andes, Ecuador, Integrative taxonomy

## Abstract

We explore the hidden diversity of the terrestrial frog genus *Niceforonia*, based on phylogenetic and morphological evidence, we describe two new species. Both species are found in pristine unexplored old-growth forest of the Andean slopes of Ecuador. *Niceforonia dracula* sp. nov., is only known from its type locality at the base of the iconic Cerro Golondrinas, located in the northwesternmost part of the Andes of Ecuador, Carchi Province. This new species is characterized by the presence of low occipital and dorsolateral folds, pale ventral coloration, greatly enlarged toe discs, and circunmarginal grooves in fingers and toes. *Niceforonia quilloturo* sp. nov. is known from several nearby localities in the upper Pastaza watershed, and is characterized by slightly enlarged tip in fingers and toes, presence of low occipital folds, plus small white pale pointy warts with dark belly and ventral surfaces. Ventral coloration is sexually dimorphic: dark brown with small white points in females and cream, yellow-orange peppered with small black flecks in males. Phylogenetic evidence is concordant with allopatric distribution in *Niceforonia dracula* sp. nov. and *N. elassodiscus*, while most of the taxa on the eastern of the Andes show parapatric distribution patterns, including *Niceforonia quilloturo* sp. nov., and related *Niceforonia peraccai*. Additional undescribed taxa are intermixed within *Niceforonia nigrovittata*, thus future systematic revision of the genus is needed.

## Introduction

Frogs of the genus *Niceforonia* Goin & Cochran (1963) are small, terrestrial, direct-developing anurans, characterized by pointed fingertips, terminal phalanges terminating in an expanded knob but not distinctly T-shaped, and relatively smooth dorsal skin. This genus has not been studied extensively and little is known about its biology, however most species are threatened with endemic distribution and new assessments are needed (IUCN, 2026; Acosta-Galvis et al., 2018). Fifteen species are currently described, across the Colombian and Ecuadorian Andes, the Eastern Andes in central Peru, and eastward into the Amazon Basin in Ecuador and northern Peru (Frost, 2025; Goin & Cochran, 1963). The genus has been subject to considerable taxonomic uncertainty since its description, it was recently merged with the genus *Hypodactylus* (Hedges, Duellman & Heinicke, 2008) and placed within the subfamily Hypodactylinae (Acosta-Galvis et al., 2018).

Although most type specimens of species in the genus *Niceforonia* are deposited in North American and Colombian museums (Frost, 2025), a significant research efforts in Ecuador over the last decade has been conducted in historical localities, as well as in pristine, remote, and mostly unexplored cloud forest along the Andean slopes (Coloma & Duellman, 2024). These efforts, resulted in the discovery of many new amphibian species, the strengthening of local museum collections, and improved understanding patterns of endemism and diversity in the Tropical Andes (Coloma & Duellman, 2024; Ron et al., 2024; Ortega, Brito & Ron, 2022; Yánez-Muñoz et al., 2024). These discoveries have also contributed significantly to the protection of threatened habitats (Ron et al., 2018; Yánez-Muñoz et al., 2021, 2025).

As a result of these field efforts, numerous specimens of *Niceforonia* have been collected over the years, resulting in a comprehensive series from across Ecuador and unveiling a remarkable increase in the known richness of the genus. Here, we integrate morphological and phylogenetic data to describe two new species discovered in two regions of the Tropical Ecuadorian Andes. The mountains of the Llanganates-Sangay Ecological Corridor in the Pastaza river valley in east central, and the Cerro Golondrinas-Dracula Reserve, near the Colombian border in the extreme northwest of the country. Both sites are known for their high levels of endemism in orchids and amphibians (Ríos-Alvear et al., 2024; Yánez-Muñoz et al., 2024; Monteros, Restrepo & Baquero, 2022).

## Materials and Methods

### Ethics statement

This research was conducted under Ecuadorian law “Código Orgánico del Ambiente-Registro Oficial N° 983”, Research permits were issued by the Ecuadorian environmental authorities in accordance with local regulations: MAE DNB CM 2019-0120, MAATE ARSFC 2023 3346, MAATE-ARSFC 2024-0847. We followed recommendations of Beaupre et al. (2004), regarding Ethical standards established by the American Society of Ichthyologists and Herpetologists, the Herpetologists League, and the Society of Study of Amphibian and Reptiles.

**Systematic & morphological features.** Terminology and diagnostic characters used for *Niceforonia* are based on the original description of the genus (Goin & Cochran, 1963), and subsequent revisions of related species (Cannatella, 1984; Lynch, 1968, 1973, 1975; Duellman, 2000). For species descriptions and diagnosis, we used amphibians terminology *sensu*, Duellman & Trueb (1994), and Duellman & Lehr (2009) for Strabomantid frogs, Diagnostic characters, definitions, and diagrams follow Lynch (1973) and Duellman & Lehr (2009). For family systematic classification we followed the criteria proposed by Heinicke, Duellman & Hedges (2007), Hedges, Duellman & Heinicke (2008), and Padial, Grant & Frost (2014).

The electronic version of this article, (PDF format) constitutes a published work according to the International Commission on Zoological Nomenclature (ICZN). Accordingly the new names contained in the electronic version are effectively published from the electronic edition alone. This published work and the nomenclatural acts it contains have been registered in ZooBank, the online registration system for the ICZN. The ZooBank LSIDs (Life Science Identifiers) can be resolved and the associated information viewed through any standard web browser by appending the LSID to the prefix http://zoobank.org/. The LSID for this publication is: [LSIDurn:lsid:zoobank.org:pub:1E0F3CA8-699C-4618-B137-53017A2560C2]. The online version of this work is archived and available from the following digital repositories: PeerJ, PubMed Central SCIE and CLOCKSS.

**Field Work:** All specimens of the new taxa were collected during field expeditions and herpetological inventories of EcoMinga, INABIO and QCAZ Research Teams. Field sampling collection techniques follow Lips et al. (2001) and are described in detail in Yánez-Muñoz, Reyes-Puig & Morales-Mite (2013), Reyes-Puig et al. (2022).

Live specimens were photographed and subsequently euthanized using benzocaine; liver and muscle tissues were extracted for downstream phylogenetic analysis. Following recommendations made by Rueda, Castro & Cortéz (2006), after tissue collection, specimens were fixed on 10% formalin for twenty-four hours and later preserved in 70% ethanol. Sex and maturity were determined by the presence of secondary sexual traits (vocal slits and nuptial pads in males, body size), and direct gonad inspection through dorsolateral incisions.

**Laboratory work:** Laboratory examinations were carried out in the herpetological collections of the Instituto Nacional de Biodiversidad (DHMECN) and the Museo de Zoología of the Pontificia Universidad Católica del Ecuador (QCAZ). Morphometric terminology and measurement protocols follow those outlined by Duellman & Lehr (2009), a standard reference widely used in anuran taxonomy of Strabomantids frogs. The variables recorded include SVL (snout–vent length), TL (tibia length), FL (foot length), HW (head width), ED (eye diameter), IOD (interorbital distance), EW (upper eyelid width), IND (internarial distance), EN (eye–nostril distance), HaL (hand length), FaL (forearm length), and TaL (tarsus length).

Digits of the hand were numbered from the preaxial to the postaxial side (I–IV). Relative lengths of Toes III and V were evaluated with reference to Toe IV, whereas the comparative lengths of Fingers I and II were determined by directly opposing them. All morphometric measurements were obtained using a digital caliper with a precision of ±0.01 mm. To ensure consistency and reduce potential measurement error, each variable was measured twice by the same observer under a stereoscopic microscope.

Geographic coordinates and elevation of type localities were recorded using Garmin GPS unit (Datum WGS 84) based on collector field notes. The following abbreviations were used for photograph recognition and related information: Juan P. Reyes-Puig (JPRP), Julio C. Carrión-Olmedo (JCC), Mario H. Yanez Muñoz (MYM), Zane Libke (ZL).

**Phylogenetic analysis.** DNA extraction, PCR amplification, and subsequent nanopore sequencing followed the same protocol as Reyes-Puig et al. (2024) and Yánez-Muñoz et al. (2025). Laboratory work was performed in Ecuador at the Biotechnology Laboratory of the Instituto Nacional de Biodiversidad (INABIO) in Quito. We targeted the mitochondrial genes 12S rRNA, 16S rRNA and ND1 for the phylogenetic inference.

We imported *Niceforonia* mtDNA sequences available in GenBank, originally made available by Zumel, Buckley & Ron (2022), Carrión-Olmedo & Ron (2021), Reyes-Puig et al. (2020), Acosta-Galvis et al. (2018), Venegas et al. (2018), and Heinicke, Duellman & Hedges (2007). We built a mtDNA character matrix in Mesquite (Maddison & Maddison, 2025) with 45 newly generated sequences and 13 sequences retrieved from GenBank. We constructed a partitioned matrix with the mtDNA. The partitions were as follows: 12S rRNA, tRNA-Val, 16S rRNA, tRNA-Leu, and codon positions in ND1. Character mtDNA Matrix was aligned using default parameters in MAFFT (Katoh & Standley, 2013).

We chose maximum likelihood as an optimality criterion. Substitution models and phylogenetic analysis were conducted using IQ-Tree 1.6.8 (Nguyen et al., 2015) under default settings on the High Performance Cluster (SI/HPC), Smithsonian Institution https://doi.org/10.25572/SIHPC.

## Results

### Phylogenetic relationships

**Figure 1:**
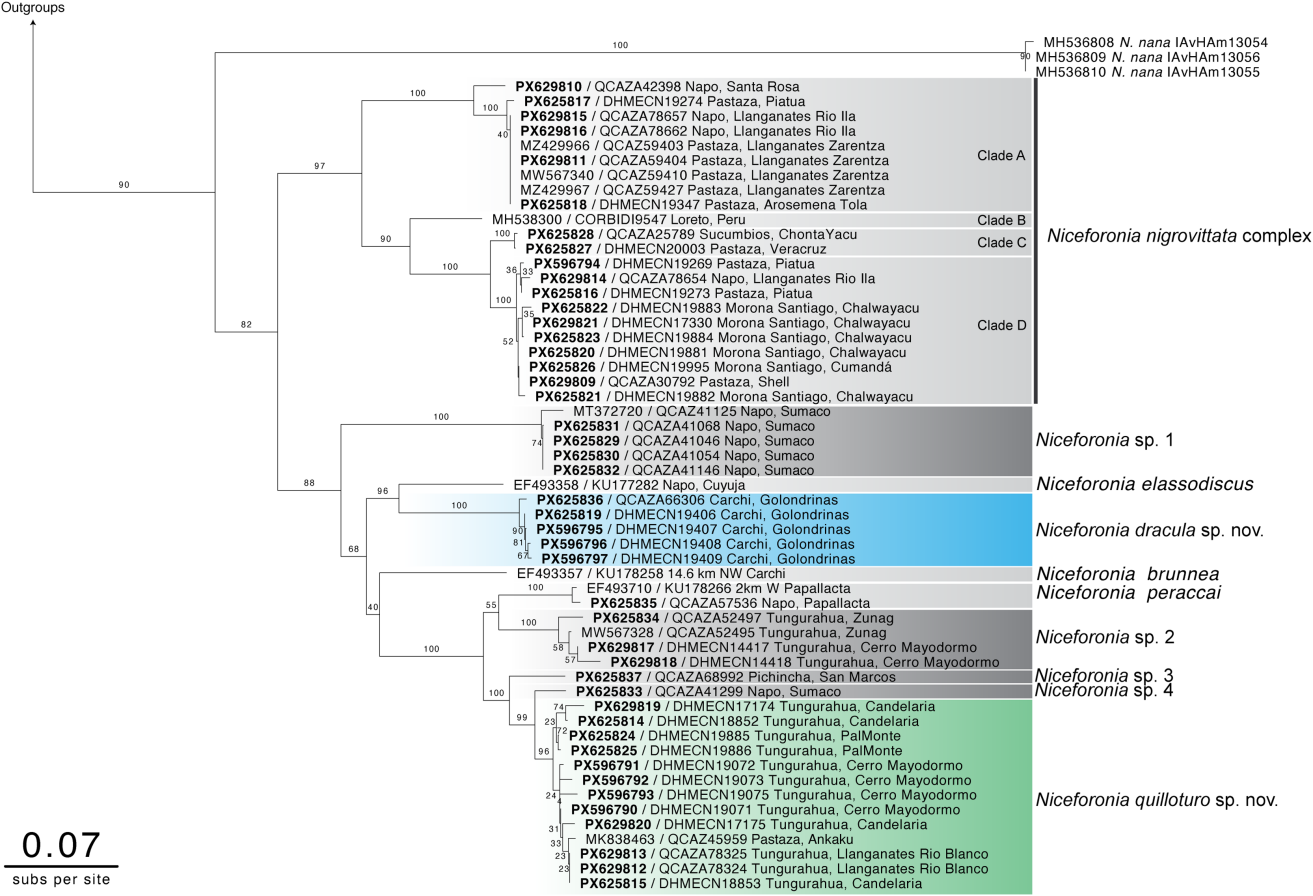
Maximum likelihood phylogenetic tree of *Niceforonia* genus based on mitochondrial sequences. Newly generated sequences are shown in black bold fonts, and GenBank accessions from previous studies are in black. Major clades of the *Niceforonia nigrovittata* complex (clade A through clade D) are highlighted in gray. *Niceforonia dracula* sp. nov. from northwestern Ecuador (Carchi, Cerro Golondrinas) are highlighted in blue, and sequences of *Niceforonia quilloturo* sp. nov. from eastern slopes of the Andes (Tungurahua and Pastaza provinces) are shown in green. Bootstrap support values are indicated next to nodes.

We increased the *Niceforonia* mtDNA sampling by more than 300%. Our phylogenetic reconstruction reveals a highly diverse assemblage within the genus, a pattern emphasized after incorporating 45 newly generated sequences from several localities along the Ecuadorian Andes. This expanded taxon sampling is in general concordant with the topology reported by Acosta-Galvis et al. (2018), but reveals several previously unrecognized lineages, of which two are formally described herein as new species (Fig. 1).

Additionally, our results suggest that *Niceforonia nigrovittata* is a species complex that comprises four clades with three candidate species. Geographically, these four clades show a latitudinal distribution across the Andes, with small range of sympatry between clade A and D. All clades within the *Niceforonia nigrovittata* complex are strongly supported (>90% branch support). Nevertheless, *N*. *nigrovittata *sensu* stricto* could not be clearly delimited until more detailed revision, indicating the need for further formal taxonomic analyses.

The sister clade to the *Niceforonia nigrovittata* complex is composed of three described species, *N. elassodiscus*, *N. brunnea*, and *N. peraccai*, plus two new species described herein, *N. dracula* sp. nov. and *N. quilloturo* sp. nov., and four candidate species (*N*. sp. 1, *N*. sp. 2, *N*. sp. 3 and *N*. sp. 4).

The earliest diverging lineage of this clade is an undescribed species (*Niceforonia* sp. 1, Fig. 1) from Sumaco, Napo Province. The second major clade comprises *N. elassodiscus* and *N. dracula* sp. nov. (bootstrap 90). *Niceforonia elassodiscus* was recovered as a strongly supported monophyletic lineage from Cuyuja, Napo Province. Its sister clade, also strongly supported (bootstrap ≥ 96), corresponds to *Niceforonia dracula* sp. nov. and includes all samples from Golondrinas, Carchi Province. This lineage is consistently recovered as monophyletic and genetically distinct from other congeners, supporting its recognition as a new species.

*Niceforonia brunnea* and *N. peraccai* were each recovered as distinct lineages, although their relationships to neighboring clades show lower support at deeper nodes (bootstraps 40 and 50 respectively). Another major, well-supported clade corresponds to *Niceforonia quilloturo* sp. nov., which includes specimens from multiple localities in the upper Pastaza watershed.

In addition to the new species described herein, the analysis recovered at least four candidate species (here referred to as *Niceforonia* sp. 1–4). These lineages are associated with discrete geographic regions, including Napo (Sumaco), Pichincha (San Marcos), and Tungurahua. However, *Niceforonia* sp. 3 and *N*. sp. 4, are each represented by a single specimen, and their phylogenetic placement should therefore be interpreted with caution. Additional sampling and integrative taxonomic analyses will be necessary to assess the species status of these candidate species.

### Systematics

**Generic Placement.** Based on phylogenetic relationships and morphological characters, we assign the two new species described in presents work to the genus *Niceforonia* Goin & Cochran (1963), characterized by moderately small frogs (SVL < 50 mm), head narrower than the body; fingers and toes lack fringes and webbing, terminal phalanges terminating in an expanded knob but not distinctly T-shaped, pointed fingertips and relatively smooth dorsal and ventral skin texture.

***Niceforonia dracula* sp. nov.**

LSIDurn:lsid:zoobank.org:act:17F177F5-7A76-4C49-BA63-503D42D2D24A

**Proposed standard English name. Dracula andean Frog**

**Proposed standard Spanish name. Sapo andino de Dracula**

**Figure 2:**
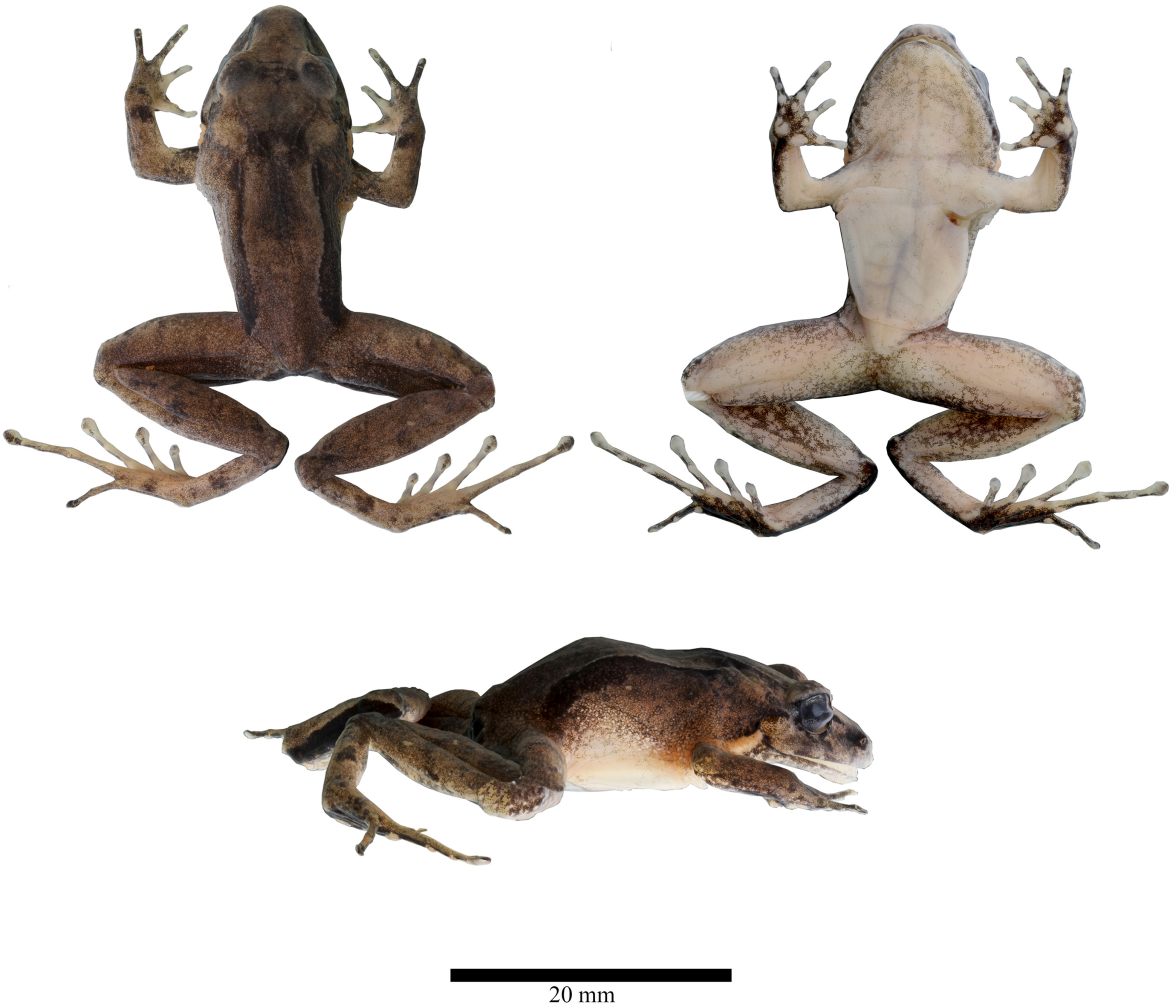
Dorsal, ventral and lateral detail of the holotype of *Niceforonia dracula* sp. nov., adult female (DHMECN 19410). Photo credit: Juan P. Reyes Puig.

**Figure 3:**
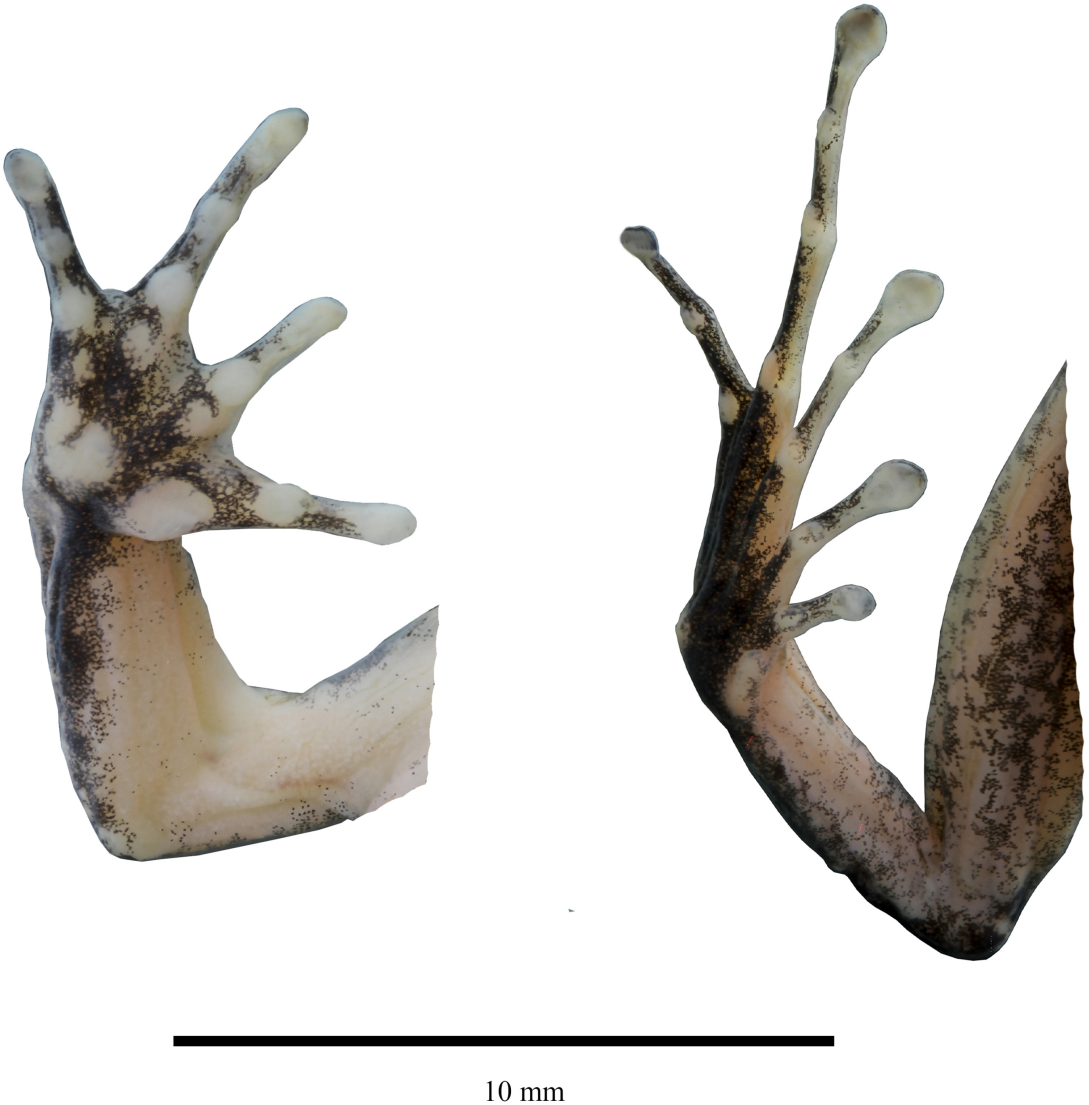
Detail of hand and feet of the holotype of *Niceforonia dracula* sp. nov., female (DHMECN 19410). Photo credit: Juan P. Reyes-Puig.

**Figure 4:**
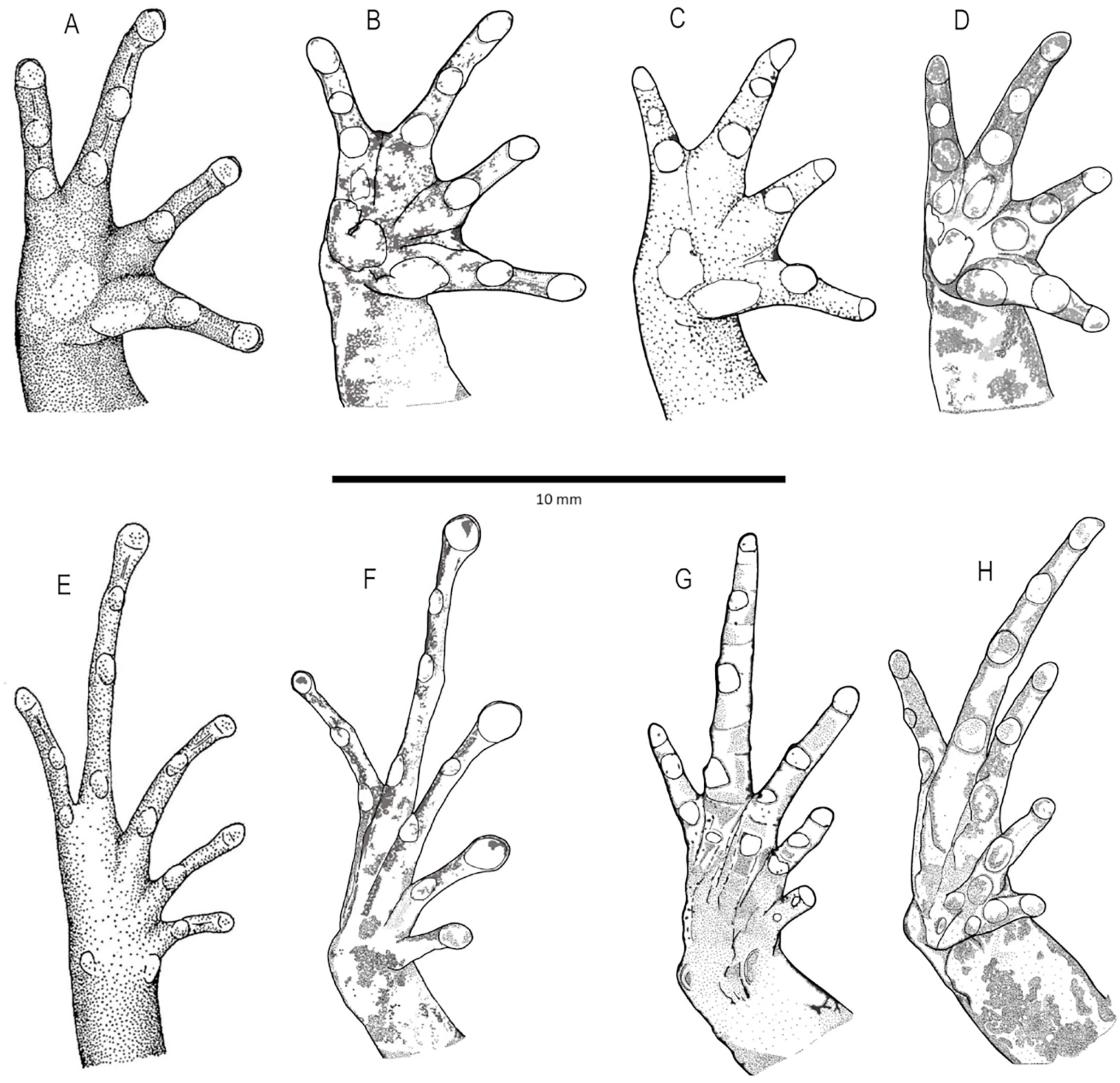
Comparison diagrams of hand and feet ventral views of new *Niceforonia* from Ecuador and related species. (A–E) *Niceforonia elassodiscus* reproduced from original description Lynch, 1973; (B–F) *Niceforonia dracula* sp. nov., holotype DHMECN 19419; (C–G) *Niceforonia peracai* KU117795 based on Lynch, 1975; (D–H) *Niceforonia quilloturo* sp. nov., holotype DHMECN 18371. Diagrams based on Lynch, 1973 (A, E) and Lynch, 1975 (C), and new species holotypes hand and feet by Miguel Urgilez-Merchán & Mario Yánez-Muñoz.

**Figure 5:**
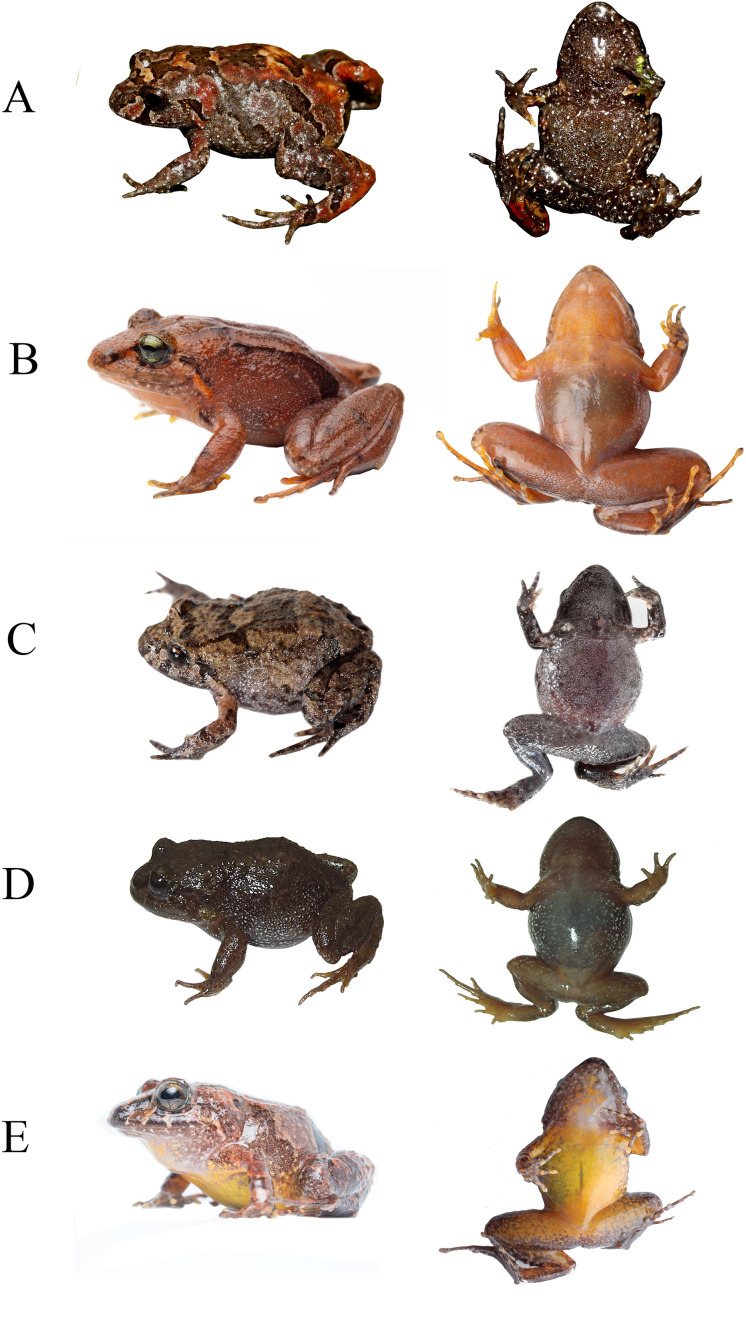
Life coloration comparison between two new *Niceforonia* in Ecuador and related species. (A) *Niceforonia quilloturo* sp. nov., DHMECN 18371, female holotype from Cerro Candelaria; (B) *Niceforonia dracula* sp. nov., DHMECN 19410, female holotype from Cerro Golondrinas-Reserva Dracula; (C) *Niceforonia peraccai* QCAZ 57537, female, topotypic from Papallacta; (D) *Niceforonia brunnea* DHMECN 6136, female from Carchi; (E) *Niceforonia nigrovittata* near type locality Abitahua. Photo credits Juan P. Reyes Puig, Callie Broaddus, Santiago Ron, Mario Yánez Muñoz & Zane Libke.

**Holotype (Figs. 2–5).** DHMECN 19410, adult female, from Reserva Dracula, Bosque Protector Cerro Golondrinas, Tulcan, Carchi Province, Ecuador, (WGS84 18N, 0.85785°N, 78.19818°W; 2,496 m), collected on 18–19 October 2023 by Juan P. Reyes Puig and Milton Cantincuz.

**Figure 6:**
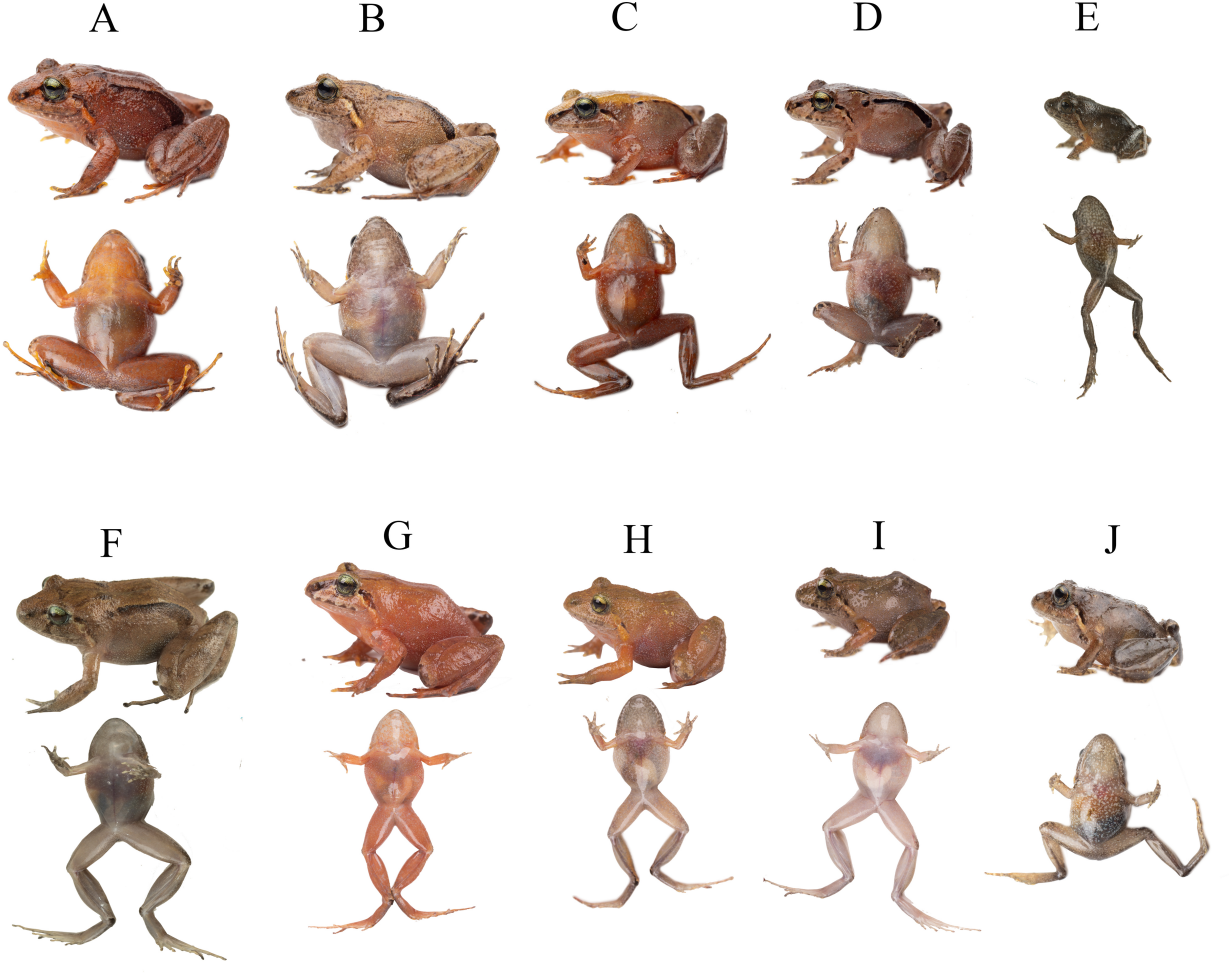
Life coloration and variation of *Niceforonia dracula* sp. nov. type series. (A) DHMECN 19410, female holotype; (B) DHMECN 19409, female; (C) DHMECN 19406, male; (D) DHMECN 19407, male; (E) DHMECN 19408, juvenile; (F) QCAZA 66299, male; (G) QCAZA 66295, female; (H) QCAZA 66305, juvenile; (I) QCAZ 66301, juvenile; (J) QCAZ 66304, juvenile. Photo credit: Callie Broaddus & Santiago Ron-Bioweb.

**Paratypes (Figs. 5, 6).** A total of sixteen (16) specimens. Adult females (8): DHMECN 19409 collected with the same dataand collectors as the holotype; QCAZA 66295-66298, QCAZA 66300, QCAZA 66303, QCAZA 66306, from Bosque Protector Cerro Golondrinas, trail to Santa Blanca, El Goaltal, Espejo, Carchi Province, Ecuador, (WGS84 18N, 0.8223799°N, 78.09228°W; 2,554 m), collected on 12–14 December 2016 by Diego Almeida, Eloy Nursirquia, Darwin Nuñez, Fernando Ayala, David Mantilla, Santiago Recalde, Carlos Castro, Polibio Malte, Josué Quintanchala; Adult Males (5): DHMECN 19406, DHMECN 19407, collected with the same type locality and data collectors of the holotype; QCAZA 66299, QCAZA 66302, QCAZA 66305, collected with the same type locality and data collectors of QCAZA 66306; Juveniles (3); DHMECN 19408 collected with the same type locality and data collectors of the holotype; QCAZA 66301, QCAZA 66304 collected with the same type locality and data collectors of QCAZA 66306.

**Diagnosis.** (1) Skin of dorsum and belly smooth; (2) low occipital ridge extending posterior and oblique from the lower border of the eyelid to the scapular region; dorsolateral fold presents or absent, extending from the scapular area to the sacrum; discoidal fold present; (3) tympanic annulus oval, tympanum hidden bellow skin, 44.51% length of eye, with thin dark supratympanic fold, glandular pale postrictal fold presents; (4) snout slightly elongated and subacuminate in dorsal view, in lateral view moderately elongated and rounded with flared lips; (5) upper eyelid width less than interorbital distance; no cranial crests; (6) vomerine odontophores prominent, transverse in outline with 9–10 serrated teeth; (7) vocal slits slightly evident; nuptial excresences presents; (8) Finger I longer than Finger II; tip of fingers not dilatated slightly elongated, presents weak circumferential grooves on digital pad; (9) fingers lack lateral fringes or keels; (10) ulnar tubercles absent; (11) heel and tarsus lacking tubercles or folds; (12) inner metatarsal tubercle elongated, about two times size of outer metatarsal tubercle that is low and rounded; no supernumary plantar tubercles; (13) toes presents thin lateral fringes and weak circunmarginal grooves, webbing absent; Toe V longer than Toe III; toes bearing expanded disks, slightly spatulated at tip; (14) dorsum orange to brown brick color, to pale brown, dorsolateral fold is delineated by dark brown or black broad line, extending from scapular to the inguinal area; throat and ventral surfaces varies from gray to orange with pale mottled, posterior surfaces of thighs light gray to orange; weak banded pattern on limbs, dark ornamentation presents on the vent and meta tarsal region; golden iris with small black reticulations and horizontal coppery stripe; (15) SVL males 19.58–23.51 mm SVL, females 26.9–34.11 mm.

**Comparison with similar species.** *Niceforonia dracula* sp. nov. differs from other *Niceforonia* species in Ecuador by the combination of occipital and dorsolateral folds present or absent, plus slightly expanded digits and greatly elongated slighthly spatulated toes with weak circunmarginal grooves (Figs. 4, 5, Table 1). All other species in montane forests of Ecuadorian Andes (*N*. *elassodiscus*, *N*. *brunnea*, *N*. *peracai*), lack’s rounded expanded tips of the digits, toes and circunmarginal grooves, nor occipital and dorsolateral folds. Also, the new species exhibit 9–10 transverse vomerine odontophores, in contrast, its sister species, *Niceforonia elassodiscus*, has 5–8 transverse vomerine odontophores and rounded tips on both fingers and toes. *Niceforonia quilloturo* sp. nov., on its turn presents slightly enlarged tips of the digits and toes and circunmarginal grooves absent, *Niceforonia elassodiscus* bear rounded tips of fingers and toes. *Niceforonia babax* and *N. philippi* on its turn presents rounded snouts, completely different from subacuminated snout of *N. dracula* sp. nov., *Niceforonia mantipa* from the western cordillera in Colombia, don’t exhibit dorsolateral or occipital folds.

**Table 1:** Diagnostic characters for two new species of *Niceforonia* in comparison with other members of the genus in Ecuador.

Reference (Holotype)	Species	Dorsolateral folds	Occipital folds	Dorsal texture	Snout dorsal and lateral profile	Digital pads	Dorsal color	Ventral color	SVL males	SVL females
This work (DHMECN 19410)	*Niceforonia dracula* sp. nov.	Presents low	Presents low	Smooth	Subacuminate dorsal, long and rounded in profile	Slightly enlarged digits, toes spatulated, thin fringes, weak circunmarginal grooves without	Dark orange, through brown to light grey coloration, with pair of black marks in scapular region, and dark dorsolateral ridges	Pale orange or gray tones with throat mottled, dark ornamentation on the vent and tarsal surfaces	19.9–27.79	27.32–34.11
This work (DHMECN 18371)	*Niceforonia quilloturo* sp. nov.	Presents low	Presents low	Smooth with scarce diminute pointed white warts	Sub acuminate dorsal, sloping in lateral view	Not dilatated slightly enlarged without circunmarginal grooves	Varies in a range on dark brown, black gray tones, in females	Gray with white small points	13.99–21.01	14.17–20.66
Lynch (1973) NMNH 167668	*Niceforonia elassodiscus*	Absent	Absent	Smooth to feebly pustulate	Subacuminate dorsal, sloping in lateral	Rounded apically	Pinkish tan to grayish brown with olive-brown to dark	Venter gray to brown, with or without silvery flecks laterally	22.0–29.2	29.7–36.9
Lynch (1975) (USNM 192909)	*Nicefornia brunneus*	Absent	Absent	Smooth	Rounded lateral	Not dilatated	Brown markings	Brown with indistinct dark mottling	25.8–27.6	26.9
Lynch (1975) (USNM 160947)	*Niceforonia peracai*	Absent	Presents	Smooth with flat wart on flank and eyelid	Rounded to truncate in profile	Not dilatated rounded apical	Gray-brown with darker brown markings	Cream spots in the venter	21.6	
Lynch & Duellman (1980) (NHRM)	*Niceforonia nigrovitatta*	Incomplete	Absent	Finely shagreened with small tubercles	Acuminated in dorsal view, pointed in males rounded in females in lateral view	Elongated discs not expanded digits	Dorsum brown with darker brown blotches;	Venter cream	17.2–24.6	25–30.5
Lynch & Duellman (1980) (KU 143505)	*Niceforonia philipi*	Absent	Absent	Pustulate with short ridges	Subacuminate in dorsal view, round un lateral profile	Long as broad pads narrow	Brown with darker blotches, posterior thigh brown with cream flecks	Dirty white reticulated with brown	35.8–40.3	56–57.6
Lynch (2003) (ICNMHN 13592)	*Niceforonia babax*	Presents incomplete	Absent	With low warts	Snout subacuminate in dorsal view, rounded to truncate in profile	Slender digits with narrow digital discs	Dorsum brown with darker brown blotches	Cream	42.4	45.6–48.7

**Description of the holotype (Figs. 3, 4).** Head broader than body; snout slightly elongated subacuminate in dorsal view, moderate in length, rounded with horizontal keel at tip of snout; nostrils protruding, directed laterally; canthus rostralis angular; loreal region slightly concave, sloping abruptly to lips; lips mottled with dark gray tones; upper lip bearing fleshy fold; upper eyelid bearing a few low flattened tubercles; interorbital space flat, broader than upper eyelid; dark supra tympanic fold obvious, obscuring upper edge of tympanic annulus which is oval in shape, tympanum hidden below skin; rough than orange glandular postrictal fold extending oblique below tympanic annulus near to the insertion of the arm; choanae small rounded, not concealed by palatal shelf of maxillary arch; vomerine odontophores median and posterior to choanae, transverse in outline, bearing a row of 9–10 acute serrated teeth; tongue longer than wide, not notched posteriorly, posterior third part not adherent to floor of mouth; short vocal slits posterolateral to tongue.

Skin of head, dorsum and flanks slightly rugose, venter smooth; occipital folds presents extending oblique below the eye to the scapular region, thin dorsolateral folds extending from scapular to sacrum area; without anal sheath; discoidal fold just anterior to groin; no ulnar folds or tubercles; thenar tubercle oval elongated, half size than bifid palmar tubercle; without supernumerary tubercles; subarticular tubercles of fingers flattened; fingers with low lateral fringes; fingers with slightly elongated discs, low circunmarginal grooves are evident, Finger III pointed, others rounded at tips, digits with circunmarginal grooves, Finger I longer than Finger II (Figs. 3, 4).

Heel and tarsus lacking tubercles or folds; inner metatarsal tubercle oval and elongated, length three times width; outer metatarsal rounded about third of inner metatarsal tubercle; sole lacking plantar supernumerary tubercles; subarticular tubercles of toes oval and elevated; toes with thin lateral fringes; all toes bearing expanded slightly spatulated discs, about twice width than digits, pads defined by circumferential grooves; Toe V reaches penultimate subarticular tubercle of Toe IV, Toe III reaches proximal border of penultimate subarticular tubercles of Toe IV (Figs. 3, 4); when flexed hindlimbs held perpendicular to sagittal plane, heels barely overlapping.

**Coloration in alcohol (Fig. 3).** Dorsal surfaces dark brown diffuse coloration in interobital area, canthus rostralis and lips with mottled pattern, thin dark brown supratimpanic stripe from posterior border of the eye, covering tympanic annulus superior and posterior, followed by a pale light orange postrictal fold that extends oblique posterior to a level in the insertion of the arm; fleshy protuberance on snout pale cream; a pair of dark brown scapular blotches are presents, dorsolateral folds are delineated by wide dark brown stripes, becoming darker at level of the groin, flanks becoming light brown; limbs brown with a diffuse banded pattern, dark brown marks on the wrists, to the Fingers III and IV, whereas banded pattern are more evident on posterior limbs; dark brown trapezoidal anal ornamentation; dark brown coloration on external metatarsal surfaces; chin and throat mottled with pale gray; immaculate cream venter with small black points scattered; shanks and ventral limbs with pale cream coloration, palms and soles heavily dark brown pigmented.

**Coloration in life (Figs. 2, 5).** Doral surfaces with a dark orange, resembling brick color, dark diffuse interorbital bar, snout, mottled canthal area and lips with diffuse dark brown marks, lower lips with minute white points; dark brown supratympanic stripes, followed posterior by a orange glandular postrictal folds; banded limbs with diffuse dark brown pattern, golden iris with black reticulation and numerous black points, with horizontal coppery bar. Chin and throat with bright orange and pink tones heavily peppered with diminutive black points; orange belly with small white points and scarce tiny black points; shanks and legs orange; palms and soles with dark brown areas, subarticular tubercles and discs with pale orange tones.

Measurements of holotype in mm: (DHMECN 19410) SVL: 27.79; TL: 16.19; FL: 15.65; HL: 11.18; HW: 11.14; EW: 2.54; IOD: 3.09; IND: 3.97; EN: 2.76; ED: 3.37; TD: 1.5; HaL: 6.94; Fal: 5.47; Tal: 8.85.

**Figure 7:**
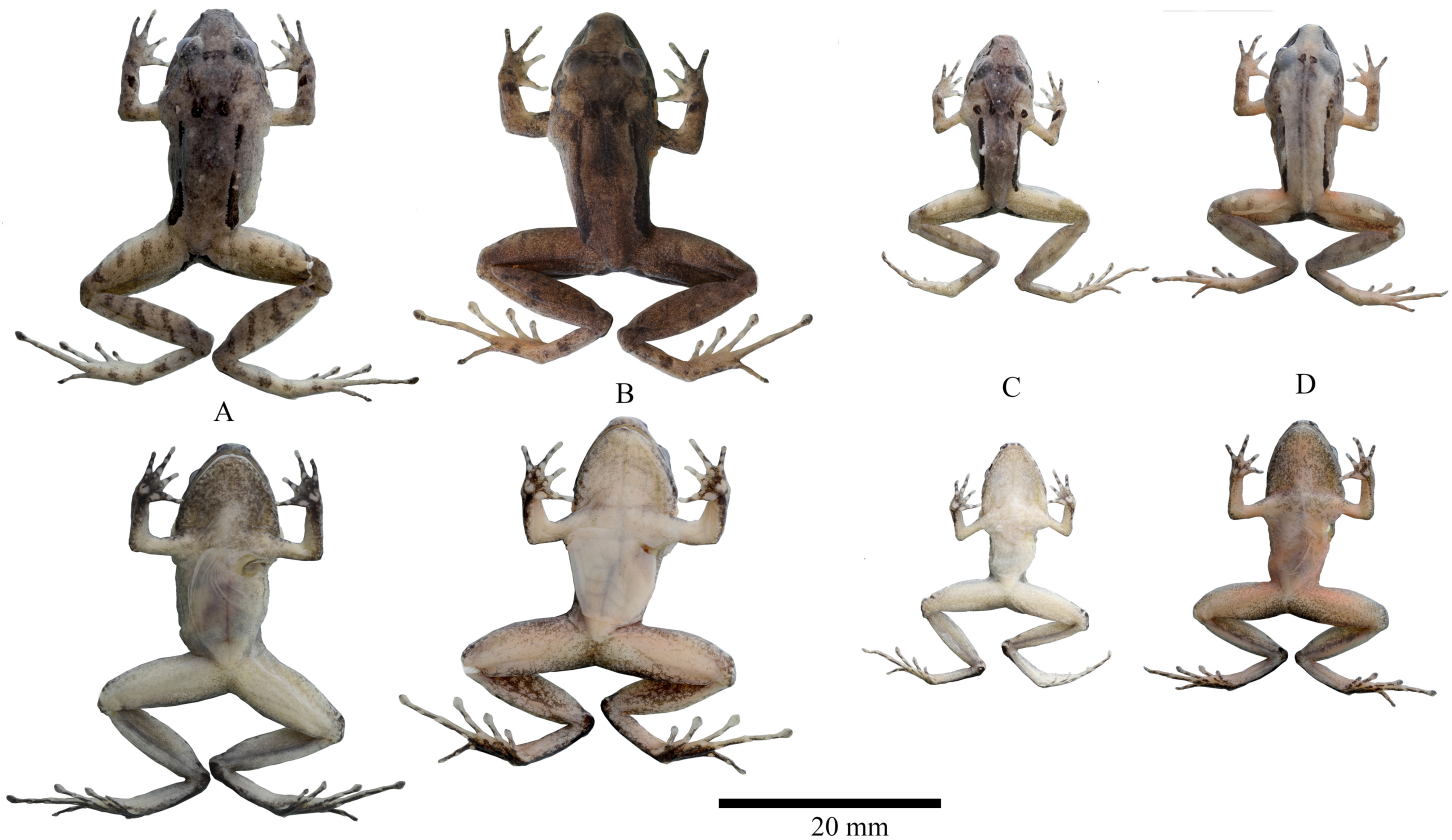
Preserved variation of *Niceforonia dracula* sp. nov. (A) DHMECN 19409, female; (B) DHMECN 19410, female holotype; (C) DHMECN 19407, male; (D) DHMECN 19406, male. Photo credits: Juan P. Reyes-Puig.

**Variation (Figs. 6, 7).** *Niceforonia dracula* sp. nov. presents sexual dimorphism in size, with larger females than males (Table 2). A wide variation on its dorsal coloration patterns, include, bright orange and yellow dorsal coloration, dorsolateral folds delineated or not with dark wide lines; dark anal ornamentation, extending to metatarsal area, dark brown diffuse marks on knees and external metatarsal areas. Ventral coloration varies from orange, brick color with white points on the belly and mottled throat in males; to the other extreme with pale grey mottled with white and dark marks.

**Table 2:** Morphological characters of two new *Niceforonia* species from unexplored mountains in Ecuador.

	*Niceforonia dracula* sp. nov.		*Niceforonia quilloturo* sp. nov.	
	Females *n* = 9	Males *n* = 5	Females *n* = 15	Males *n* = 13
SVL	26.9–34.11 (30.51 ± 5.1)	19.58–23.51 (21.55 ± 2.78)	14.17–22.41 (18.29 ± 5.83)	13.73–21.01 (17.37 ± 5.15)
HW	10.65–13.27 (11.96 ± 1.85)	7.75–9.01 (8.38 ± 0.89)	5.33–7.15 (6.24 ± 1.29)	4.89–7.61 (6.25 ± 1.92)
HL	9.19–12.06 (10.63 ± 2.03)	6.91–8.35 (7.63 ± 1.02)	5.09–7.71 (6.4 ± 1.85)	4.62–6.61 (5.615 ± 1.41)
En	2.74–3.67 (3.21 ± 0.66)	1.84–2.53 (2.19 ± 0.49)	1.22–1.87 (1.55 ± 0.46)	1.13–1.77 (1.45 ± 0.45)
IND	3.34–3.97 (3.66 ± 0.45)	2.5–2.99 (2.75 ± 0.35)	1.58–2.22 (1.9 ± 0.45)	1.39–2.22 (1.805 ± 0.59)
IOD	2.85–3.38 (3.12 ± 0.37)	2.07–2.63 (2.35 ± 0.4)	1.58–2.14 (1.86 ± 0.4)	1.62–2.27 (1.945 ± 0.46)
EW	1.96–2.63 (2.3 ± 0.47)	1.55–2.13 (1.84 ± 0.41)	0.94–1.7 (1.32 ± 0.54)	0.93–11.5 (6.215 ± 7.47)
TD	1.28–1.64 (1.46 ± 0.25)	0.78–1.07 (0.93 ± 0.21)	0.58–1.02 (0.8 ± 0.31)	0.47–1.13 (0.8 ± 0.47)
ED	2.86–3.37 (3.12 ± 0.36)	2.06–2.54 (2.3 ± 0.34)	1.48–2.31 (1.9 ± 0.59)	1.53–2.49 (2.01 ± 0.68)
TL	15.25–18.94 (17.1 ± 2.61)	10.4–13.48 (11.94 ± 2.18)	6.28–8.67 (7.48 ± 1.69)	6.06–8.97 (7.515 ± 2.06)
HaL	6.13–7.58 (6.86 ± 1.03)	4.3–5.15 (4.73 ± 0.6)	3.23–4.73 (3.98 ± 1.06)	3.11–4.5 (3.805 ± 0.98)
FL	14.67–17.49 (16.08 ± 1.99)	9.68–12.14 (10.91 ± 1.74)	4.09–10.15 (7.12 ± 4.29)	6.2–9.56 (7.88 ± 2.38)

**Note:**

Abbreviations in the Methods section.

**Etymology.** The specific epithet *dracula* is a noun in apposition (Art. 11.9.1.2 of the ICZN) chosen in reference to the region where the species was discovered, near the Dracula Reserve in northwestern Ecuador. The reserve is an area of high endemism and with unique ecological conditions. It is located in the province of Carchi, Ecuador. Reserva Dracula protects 4,000 hectares of Tropical Andes cloud forest, covering an elevation range of 900–2,460 m and is threatened by mining. Dracula reserve is named for its high concentration of orchids of the genus *Dracula*. In addition, the name evokes the misty environment where the frog lives.

**Natural history.** *Niceforonia dracula* sp. nov. has been found active at night in the thick leaf litter of mature forest dominated by *Clusia* trees, abundant moss, and epiphytes (orchids, ferns and Gesneriads). Sympatric species found with the new species include *Bolitoglossa* sp., *Phyllonastes cerrogolondrinas, Pristimantis hectus, Pristimantis calcarulatus, Pristimantic verecundus*, and *Epicrionops* cf. *bicolor*.

**Figure 8:**
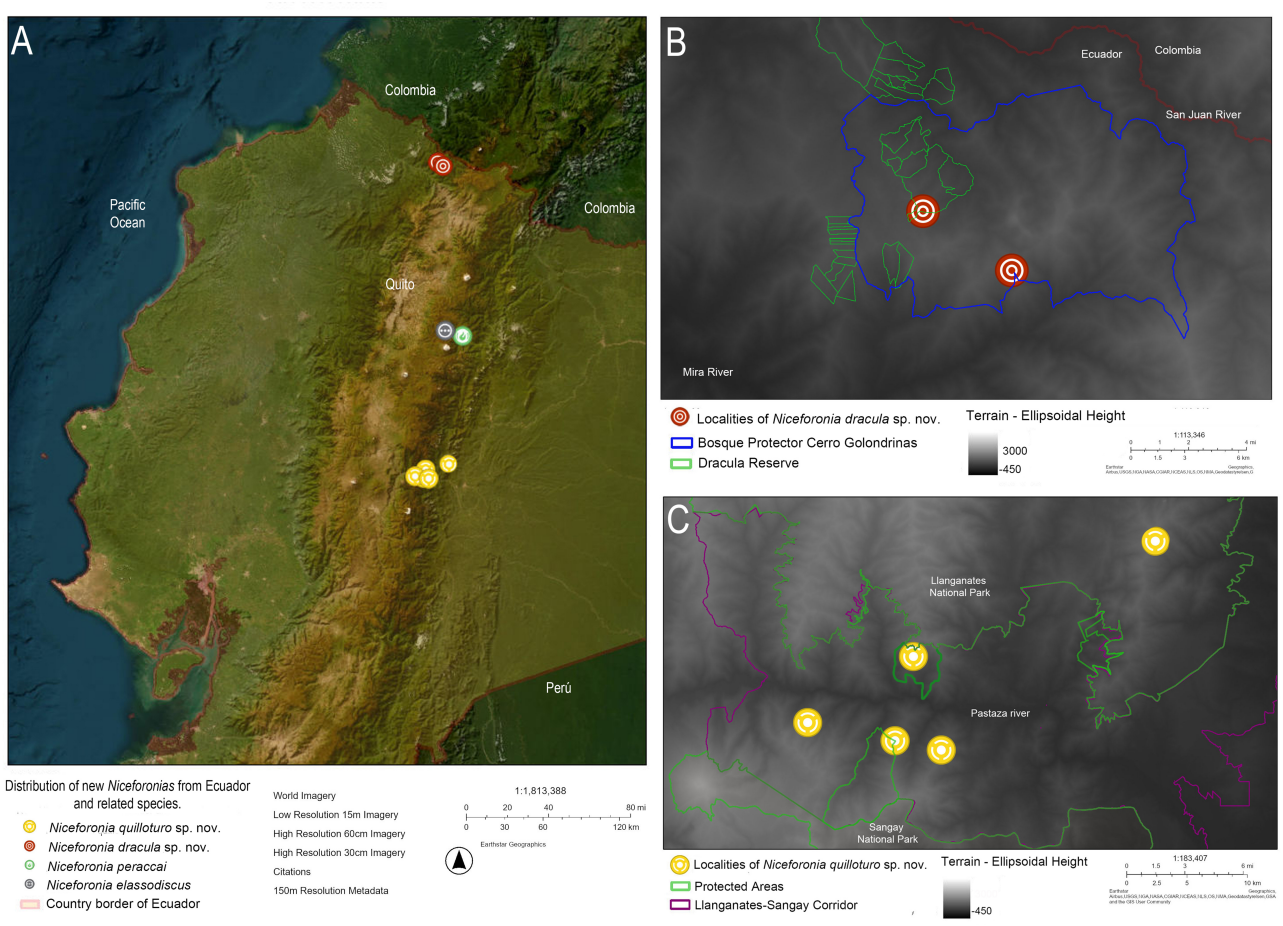
Distribution map of two new *Niceforonia* species and its closest related congeners in Ecuador. (A) General distribution of the new species and sister species in Ecuador; (B) distribution of *Niceforonia dracula* sp. nov.; (C) distribution of *Niceforonia quilloturo* sp. nov. Map by Juan P. Reyes-Puig. Base map use Earthstar Geopraphics, World Imagey 150 m Resolution Metadata.

**Distribution.** *Niceforonia dracula* sp. nov. is known only from its type locality in the northern-western slopes of Cerro Golondrinas and Dracula Reserve at extreme north western Ecuador, Carchi province, from 2,495 to 2,554 m in high Montane forest (Fig. 8).

**Conservation.** The type locality is located inside Bosque Protector Cerro Golondrinas, part of the mosaic of protected areas managed by Dracula Reserve. Despite formal protection, mining concessions persist inside and near these areas, increasing demand for mined resources places this population at serious risk in the future. We propose that the species be listed as Data Deficient (DD) under the IUCN red list until new explorations have been done in the area, especially on the northern and western slopes of the Cerro Golondrinas. Nonetheless, the species restricted range and dependence on mature forest habitats indicate a potential risk of being classified as threatened in future assessments.

***Niceforonia quilloturo* sp. nov.**

LSIDurn:lsid:zoobank.org:act:21F4FE0C-4D5A-460B-B9C6-18BD639E9DE0

***Proposed standard English name. Muddy narrow toed frog***

***Proposed standard Spanish name. Sapo de lodo de dedos angostos***

**Figure 9:**
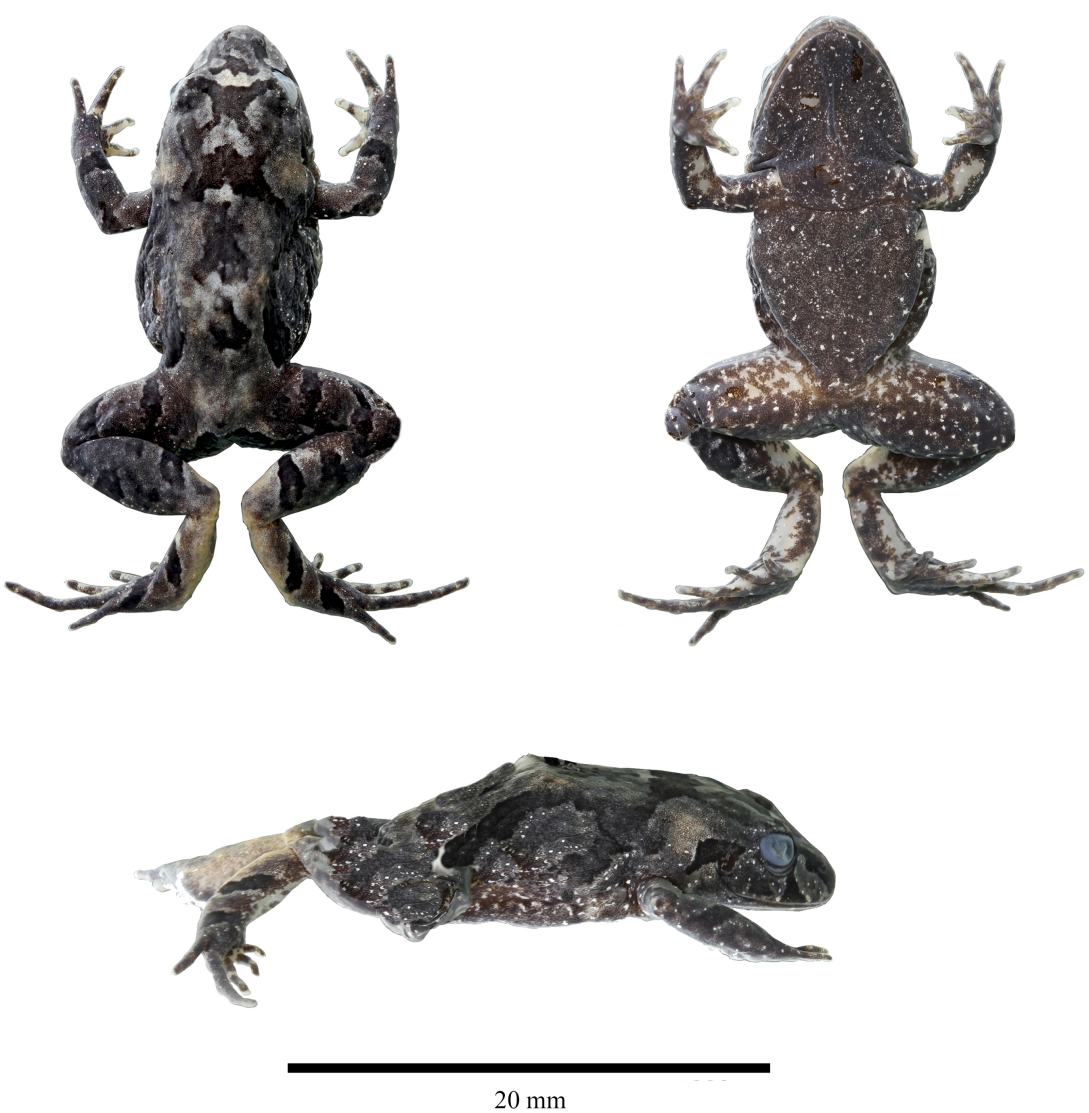
Dorsal, ventral and lateral detail of the holotype of *Niceforonia quilloturo* sp. nov., female (DHMECN 18371). Photo credit: Juan P. Reyes-Puig.

**Figure 10:**
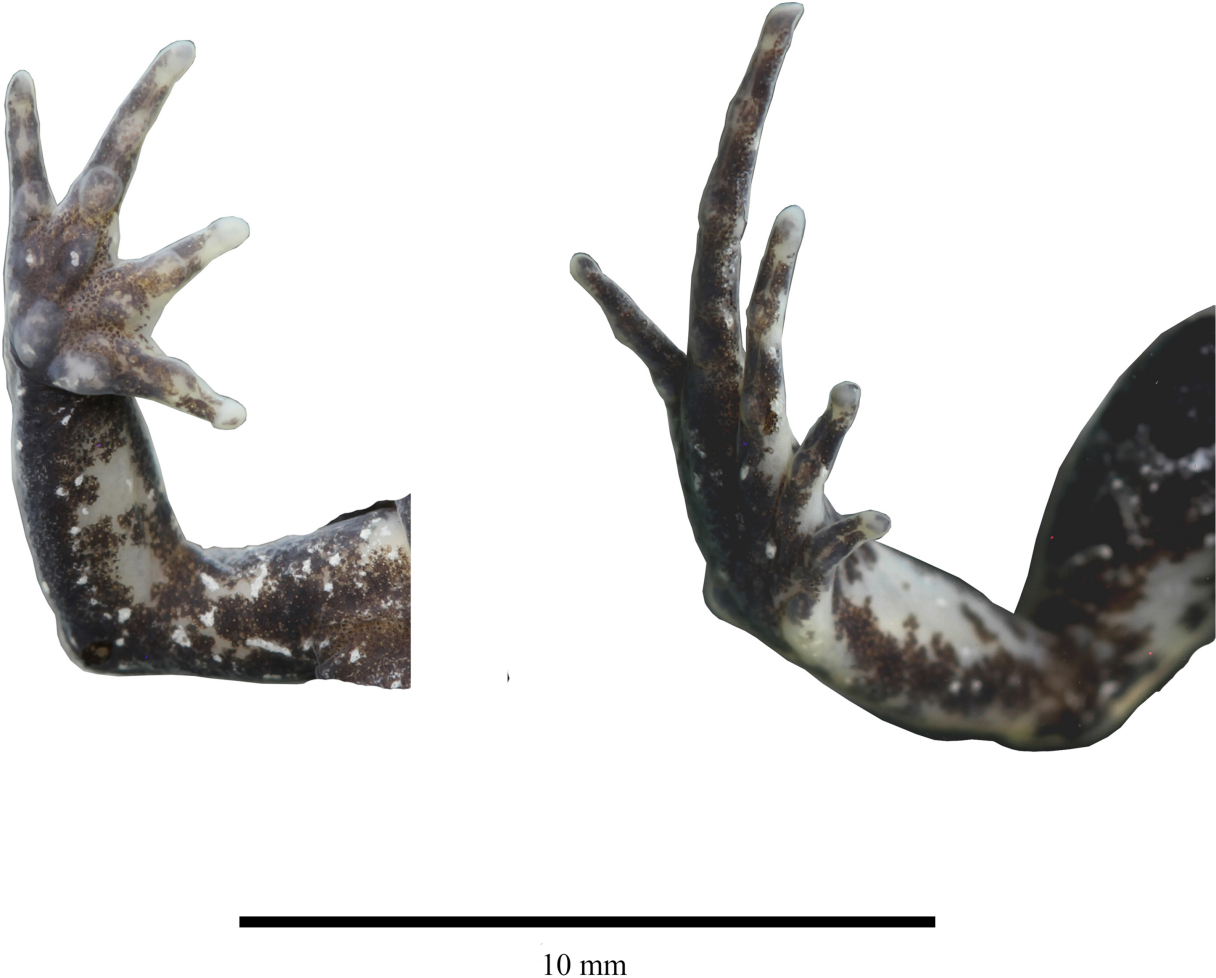
Detail of hand and feet of *Niceforonia quilloturo* sp. nov., holotype female (DHMECN 18371). Photo credit: Juan P. Reyes-Puig.

**Holotype (Figs. 9, 10).** DHMECN 18371, adult female, from Cerro Candelaria Protected Area, Baños de Agua Santa, Tungurahua Province, Ecuador, (WGS84 17N, 1.43692°S, 78.301031°W; 2,569 m) collected by Juan P. Reyes Puig, Paulet Benavides, Nantar Kuja & Patricio Vinueza on 22 November 2022.

**Figure 11:**
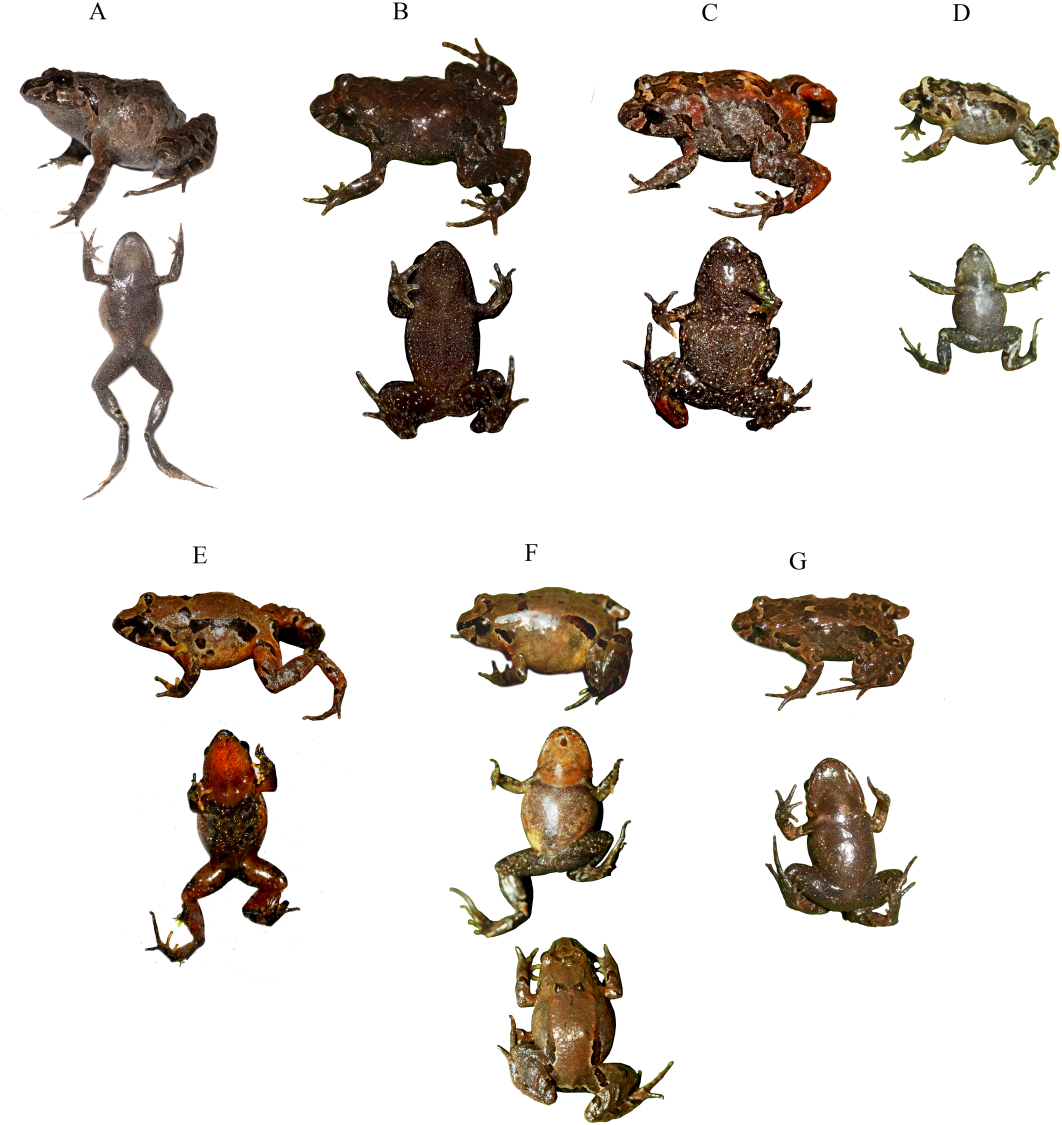
Life coloration and variation of *Niceforonia quilloturo* sp. nov. (A) QCAZA 78325, female; (B) DHMECN 18374, female; (C) DHMECN 18371, female holotype; (D) DHMECN 17176, male; (E) DHMECN 18143, male; (F) DHMECN 15255, female; (G) DHMECN 19075, male. Photo credits: Bioweb & Juan P. Reyes-Puig.

**Figure 12:**
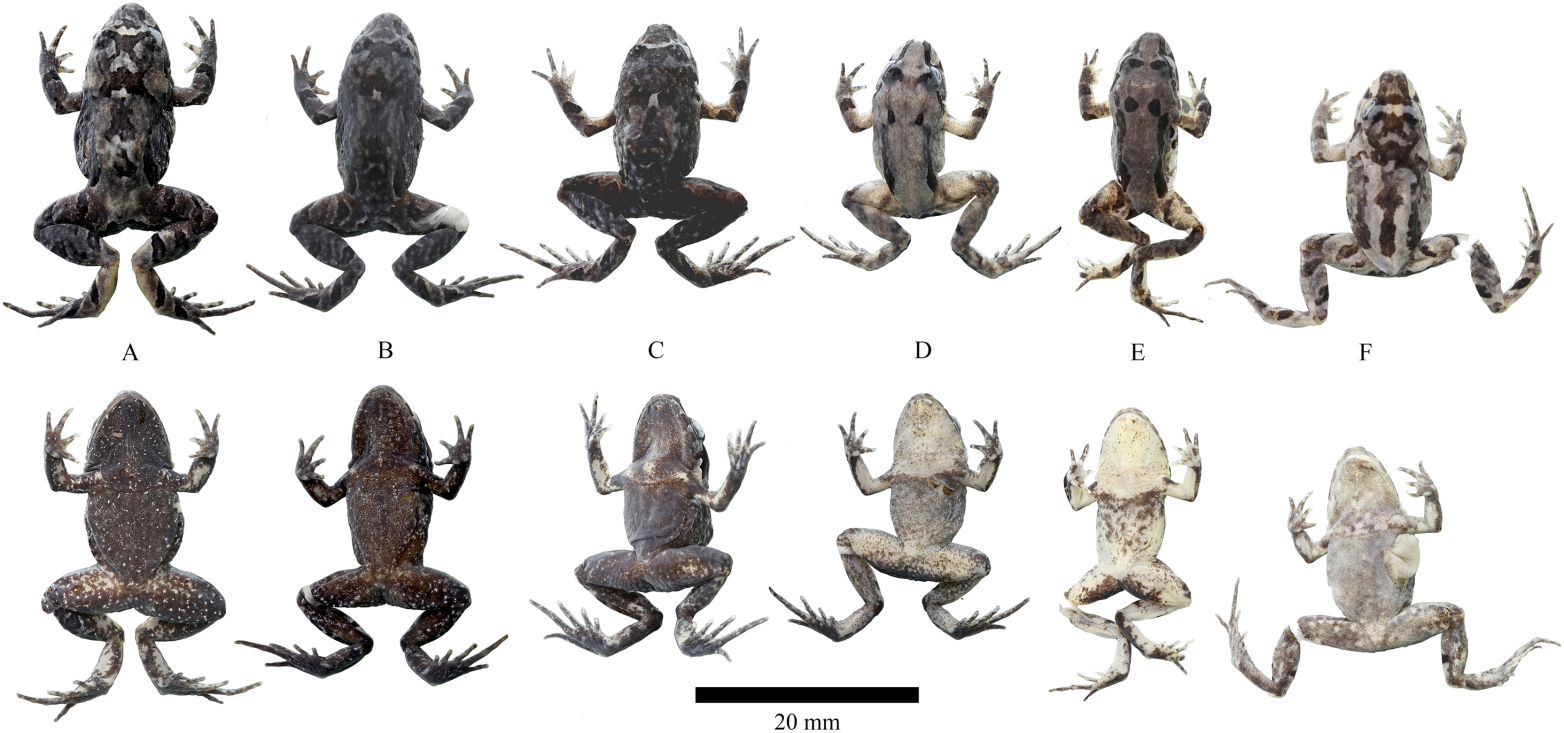
Preserved variation of *Niceforonia quilloturo* sp. nov. (A) DHMECN 18371, female holotype; (B) DHMECN 18372, female; (C) DHMECN 19075, male; (D) DHMECN 18375, male; (E) DHMECN18379, male; (F) DHMECN 17176, male. Photo credit: Juan P. Reyes Puig.

**Paratypes (Figs. 11, 12).** A total of (22) specimens. Adult females (12): DHMECN 17175-DHMECN 17176, collected in the same type locality at higher elevation than the holotype (WGS84, 17N −1.45692 S, 78.30314 W; 3,048 m) collected by Juan P. Reyes Puig and Zane Libke on 21 January 2021; DHMECN 18372, DHMECN 18374, DHMECN 18376, DHMECN 18377 DHMECN18378 collected in the same type locality and data collectors of the holotype; DHMECN 19072, DHMECN 19074 from Machay Reserve, Cerro Mayordomo, RioVerde, Baños de Agua Santa, Tungurahua province, Ecuador (1.37468°S, 78.28722°W; 2,746 m) collected by Juan P. Reyes Puig, Paulet Benavides, José Segovia & Martín Morales on 2 October 2023; DHMECN 19385, collected near the same type locality of the holotype (WGS 84, 17S 1.43988°S, 78.30508°W; 2,550 m) on 5 November 2023 by Juan P. Reyes Puig; QCAZA 78324–25, from Río Blanco, Ulba, Baños de Agua Santa, Tungurahua province, Ecuador (WGS 84 17S, 1.36364°S, 78.264247°W; 2,747 m) collected by Jhael Ortega, Fernando Ayala, Santiago Guamán, Gabriel Alejandro, Doménica Aguirre, Erick Troncoso, Emilio Valdiviezo on 25 February 2023; Adult males (10): DHMECN 17174 collected with the same data and collectors of DHMECN 17176; DHMECN 18142-DHMECN 18143, from Bosque Protector y Vegetación Protectora Guamag, Ulba, Baños de Agua Santa Tungurahua Province, Ecuador (WGS84, 17S, 1.42348°S, 78.36608°W; 2,747 m) collected by Juan P. Reyes Puig, Paulet Benavides, Nantar Kuja & Patricio Vinueza on 18 May 2022; DHMECN 18373, DHMECN 18379 collected with same locality and collection data than the holotype; DHMECN 18853; DHMECN 19071, DHMECN 19073, DHMECN 19075 collected with the same data collection than DHMECN 19072; QCAZA78323, collected with the same data and collectors than QCAZA 78325; QCAZA 45959, Ecuador, Pastaza Río Challuwa Yacu, Reserva Privada Ankaku, buffer zone Parque Nacional Llanganates, collected by Elicio Tapia on January 2009 (WGS84, 17N 1.279239°S, 78.07790°W, 2,300 m).

**Diagnosis.** (1) Skin of dorsum smooth with low rounded small tubercles on the flanks, belly smooth; (2) low occipital ridge extending posterior and oblique from the eyelid to the scapular region, a pair of rounded tubercles are presents on scapular region; dorsolateral fold or row of dorsolateral rounded tubercles presents, extending along flanks to the sacrum; discoidal fold present; (3) tympanic annulus round, tympanum hidden below skin 42.02% length of eye, thick dark supratympanic fold extending posterior to the eye and parallel to a glandular pale postrictal fold; (4) snout subacuminate in dorsal view, in lateral view moderate in length curved anteroventrally, with a fleshy flap in males; (5) upper eyelid width less than interorbital distance; no cranial crests; (6) vomerine odontophores prominent, oblique or arched in outline with 4–5 serrated teeth; (7) vocal slits slightly evident posterior and lateral to the tongue; nuptial excrescences absent; (8) Finger I equal in length than Finger II; tip of fingers not dilatated slightly elongated, lack circumferential grooves or digital pads; (9) fingers lack lateral fringes or keels; (10) ulnar tubercles absent; (11) heel and tarsus lacking tubercles or folds; (12) inner metatarsal tubercle elongated, slightly longer than outer metatarsal tubercle that is low and rounded; no supernumerary plantar tubercles; (13) toes lack lateral fringes and circunmarginal grooves, webbing absent; Toe V equal in size than Toe III; toes lacking expanded disks, tip of toes slightly elongated; (14) dorsum dark brown to pale orange, with a pair of dark brown scapular and inguinal elongated dark marks, mixed with irregular dark and pale marks, small white points scattered on the flanks, dark brown blotch on forearm, shanks and feet, dark brown anal ornamentation; throat orange to dark brown and grey, with small white points, venter brown, dark brown, to gray with small white points; posterior surfaces of thighs gray, brown to orange; copper iris with small black points; limbs presents a weak banded pattern, ornamentation presents on the vent and meta tarsal region; (15) SVL males 13.73–19.47 mm, SVL females 14.17–22.41 mm.

**Comparison with similar species (Figs. 4, 5)**. *Niceforonia quilloturo* sp. nov. differs from other *Niceforonia* in Ecuador by the combination of occipital and dorsolateral folds, plus thick dark supratympanic, stripe contrasting with a pale parallel postrictal fold; not elongated fingers or toes, not expanded disks but slightly elongated. The new species is similar to *N. elassodiscus*, whereas the last one, presents rounded digital termination and cream venter, in the *N. quilloturo* sp. nov., presents slightly elongated digits and brown or dark brown to gray venter with white small points and mottling, in *N. dracula* sp. nov., dilated pads with circunmarginal grooves and gray to orange ventral coloration are very distinctive (Table 1).

*Niceforonia quilloturo sp. nov.* exhibits oblique or arched vomerine odontophores, whereas its sister species, *Niceforonia peraccai*, shows 0–7 transverse vomerine odontophores and more acute tips on fingers and toes. Other species in eastern Andes like *N. brunnea* or *N. peraccai*, presents dark bellies and rounded digital terminations and dorsolateral fold absent, without white points on flanks and belly or elongated digital terminations and dorsolateral folds presents like in new species; *N. nigrovittata* may presents dorsolateral folds, but its ventral coloration is pale yellow gray.

*Niceforonia babax* and *N. philippi* on its turn presents rounded snouts in profile, and occipital and dorsolateral folds absent, different from new species that bears a subacuminated snout in profile plus occipital and dorsolateral folds presents.

**Description of the holotype (Figs. 9, 10).** Head wider than body; snout subacuminate in dorsal view, moderate in length, rounded in lateral and slightly projected anteriorly view; nostrils not protuberant, directed dorsolaterally; canthus rostralis rounded; loreal region slightly concave, sloping gradually to lips; upper lip bearing fleshy fold; upper eyelid bearing a few low tubercles; interorbital space flat, broader than upper eyelid; thick supratympanic fold, obscuring upper edge of tympanic annulus which is round in shape, tympanum visible; pale glandular rough postrictal fold extending oblique articulation of the jaw near the insertion of the arms; choanae small oval, not concealed by palatal shelf of maxillary arch; vomerine odontophores median and posterior to choanae, oblique in outline, bearing a row of 4–5 teeth; tongue longer than wide, not notched posteriorly, posterior third part not adherent to floor of mouth; without vocal slits.

Skin of head, dorsum and flanks slightly rugose, venter smooth; low occipital folds presents, in preservative slightly evident, low dorsolateral folds aligned with low rounded tubercles extending from scapular to sacral area; without anal sheath; discoidal fold just anterior to groin; no ulnar folds, a row of small rounded tubercles is presents; thenar tubercle oval elongated, half size than bifid palmar tubercle; without supernumerary tubercles; subarticular tubercles of fingers flattened; fingers without lateral fringes; fingers without digital dilatations or pads, but slightly elongated terminations, without circunmarginal grooves, Finger III pointed, others slightly elongated at tips, Finger I equal in length than Finger II.

Heel with 2–3 small low rounded tubercles, tarsus presents a row of small low rounded tubercles; inner metatarsal tubercle oval and elongated, slightly longer than outer metatarsal that is rounded; sole lacking plantar supernumerary tubercles; subarticular tubercles of toes oval and flattened; toes without lateral fringes; all toes without digital pads or dilatation, terminations slightly elongated without circumferential grooves; Toe V doesn’t reaches penultimate subarticular tubercle of Toe IV, Toe III reaches penultimate subarticular tubercles of Toe IV; when flexed hindlimbs held perpendicular to sagittal plane, heels barely overlapping.

**Coloration of the holotype in ethanol 70% (Figs. 9, 10).** Dorsal surfaces dark brown with pale brow tones, banded pattern and chevrons are evident along dorsal surfaces and limbs, dark bars are delineated by pale lines, interorbital pale bar followed posterior by dark thick “X” mark with irregular borders, blackish canthal and subocular stripes, small white points on the lips; thick black supratimpanic stripe extending below the eye, covering superior portion of tympanic annulus, directed posterior and oblique to the level of the insertion of the arm, followed by a pale orange postictal fold; large black “moth” shape mark on occipital region, delineated by pale tones, a medial black chevron is present at mid dorsum; flanks and limbs with dark brown banded pattern, large inguinal elongated black marks are delineated by pale lines, lower flanks dark brown with scattered white small points; limbs, fingers, and toes with a dark banded pattern, blackish bands are delineated by pale lines and light brown interspaces; dark brown oval anal ornamentation continues to the posterior surfaces of the thighs; all ventral surfaces dark brown almost blackish peppered with small white points, palms and soles with heavily dark pigmented.

**Coloration in life of the holotype (Figs. 5, 11).** Dorsal surfaces with a irregular marks pattern consisting of a dark brown blackish background, mixed with irregular cream and reddish to wine marks; dark black elongated inguinal marks delineated by pale white line; banded pattern on limbs and flanks, with dark bands delineated by pale white lines, brown interspaces mixed with scattered small white points; pale white cream interorbital bar, snout, canthal and subocular stripes black delineated by pale white lines, lips scattered with white small points; thick black supratimpanic stripe, aligned parallel inferior with a pale reddish postrictal fold with white points; limbs with blackish banded pattern delineated by pale white lines and light brown with reddish interspaces; iris copper white small black points; dark brown blackish. Ventral surfaces, heavy peppered with white small points.

Measurements of holotype in mm: (DHMECN 18371) SVL: 20.66; TL: 7.79; FL: 8.47; HL: 6.75; HW: 6.65; EW: 1.23; IOD: 1.99; IND: 1.8; EN: 1.41; ED: 1.88; TD: 0.79; HaL: 4.07; Fal: 3.86; Tal: 4.89.

**Variation (Figs. 11, 12).** Measurements range and averages of *Niceforonia quilloturo* sp. nov. are shown in Table 2, females are slightly larger than males, *Niceforonia quilloturo* sp. nov. is a highly polymorphic species on its dorsal pattern including melanic, banded, and irregular spotted patterns. Including a range of color tones from dark brown-blackish to gray, orange and brick brown color, with thick, elongated, blackish inguinal marks; Ventral colorations varies from blackish brown peppered with small white points, through gray with white small points, and extreme orange throat and ventral limbs, dark brown chest and belly with white small points scattered (Figs. 11, 12).

**Etymology.** *Niceforonia quilloturo* sp. nov. is a recognition to the town near type localities in the upper Pastaza watershed, near Rio Verde, Quilloturo was affected by two consecutive landslides that force local community of El Placer to evacuate their hometown. The new species is named in honor all the people who lost their lives and were displaced by the impacts of climate change during the rainy season of June 2024. We use the noun quilloturo in apposition, in accordance with Art. 11.9.1.2 of the ICZN.

**Natural history.** *Niceforonia quilloturo* sp. nov. is frequently found in the leaf litter of mature forest dominated by trees of the genus *Clusia*, *Oreopanax Weinmannia*, and palms of the genus *Ceroxylum* and *Geonoma*. These forests have abundant moss and epiphytic orchids, ferns, and gesneriads typical of a high Andes cloud forest. Most individuals have been encountered while active at night. Gravid females and juveniles were found along the year, suggesting different reproduction cycles. Call unknown. Sympatric Anuran species found in night surveys in southern Pastaza populations of Cerro Candelaria and Finca Palmonte include *Osornophryne simpsoni*, *Pristimantis tungurahua*, *P. marcoreyesi*, *P. normaewingae* and *P. donnelsoni*, whereas in northern Pastaza populations with *Pristimantis burtoniorum* and *P. kayi*.

**Distribution.** The new species is known from 6 localities of cloud forest within the Llanganates-Sangay Ecological Corridor, between 2,300 to 3,048 m (Fig. 8). It is found on both sides of the Rio Pastaza in Tungurahua and Pastaza provinces, northern populations (Machay, Cerro Mayordomo, Reserva Ankaku) and southern populations (Cerro Candelaria, Guamag, and Finca Palmonte). All localities where the species has been found in Evergreen montane forest of the eastern Andes under national ecosystem classification (Ministerio del Ambiente del Ecuador (MAE), 2013). All specimens were found active during night and occasionally on the day in the leaf litter of the forest.

**Conservation.** All known specimens have been found in protected areas. Cerro Mayordomo and Ankaku reserves are in the buffer zone of Llanganates National park, while Guamag, Cerro Candelaria and Finca Palmonte are in the Buffer zone of Sangay National Park. Based on the rarity of records we suggest Data deficient DD, under IUCN red list category, however the small distribution range and dependence on mature forest habitat may qualify it as a threatened species in a future assessment.

## Discussion

The present study clarifies relationships within the genus *Niceforonia* by incorporating new populations and species into the tree, generating a more complete approximation to the phylogenetic diversity hypothesis of the genus (Fig. 1), including seven species formally in Ecuador (*N. dracula* sp. nov., *N. brunnea*, *N. elassodiscus*, *N. nana*, *N. nigrovittata* complex Clade D, *N. peraccai*, *N. quilloturo* sp. nov.), and seven candidate undescribed species (*N*. sp. 1, *N*. sp. 2, *N*. sp. 3, *N*. sp. 4, *N. nigrovittata* complex Clade A, *N. nigrovittata* complex Clade B and *N. nigrovittata* complex Clade C). Coloma & Duellman (2025), mention some morphological discrepancies in taxonomic identity in some individuals and records of several taxa (*N. brunnea, N. nigrovittata, N. elassodiscus*), but beeper relationships remain unsolved due to the lack of genetic data for several populations.

Our analysis revealed an unexpectedly high diversity, including the recognition of two new species described herein, and at least four candidate new species plus a cryptic *Niceforonia nigrovittata* complex composed of four independent lineages (Fig. 1). The geographical origin of the new and candidate species, reveal several centers of endemism concordant with other amphibian and threatened biological groups already recorded along Ecuadorian Andes (Gluesenkamp, 1995; Reyes-Puig et al., 2025; Yánez-Muñoz et al., 2025; Coloma & Duellman, 2025; Ron et al., 2018).

Detailed examination of the type material photographs of *N. nigrovittata* provided by Paul Szkely from Swedish Museum of Natural History, plus fresh collected material from the same geographic area (Mera Abitagua according with original description, Andersson, 1945), provide us enough reference discriminate topotypic foothill populations (Clade D in our phylogeny is *N. nigrovittata sensu* stricto, based in dorsal and ventral coloration patterns), and other three undescribed taxa with differences in geographic and coloration patterns (Clades A, B and C).

From a biogeographic perspective, our phylogeny reveals a predominant irradiation east of the Andes across most clades (Fig. 1). This is particularly evident in *Niceforonia nigrovittata* complex, comprising 4 taxa from northern to southern Ecuador (Clades A, C, D), to a geographically isolated Clade B in north-eastern Peru. While the main diversity is concentrated in the eastern slopes of the Ecuadoriam Andes. *N. dracula* sp. nov. is the exception, representing a unique high Andean species of the clade in the western cordillera in Ecuador, *N. babax* occurs in lower elevations without genetic position available. *N. dracula* sp. nov. is the sister taxon of *N. elassodiscus* from eastern slopes in Cuyuja in the Quijos river valley, may reflect allopatric distribution in the clade, we are not aware of other reported *N. elassodiscus* populations (*e.g*., Putumayo, Colombia) until a genetic position will be assigned.

All species in the terminal branch of our topology reflects parapatric distributions along eastern slopes of the Andes in Ecuador, with *N. brunnea* in the extreme northern at Carchi in the border between Ecuador and Colombia, and *N. quilloturo* sp. nov. in the south at the Llanganates Sangay Corridor, represents geographic extremes of the clade.

Although geographic isolation plays an important role in the speciation process, ecological isolation can also exert strong influence (Magalhães et al., 2022). In the Andes, geographic barriers such as the western and eastern Cordilleras, hydrographic domains, including the Pastaza and Napo rivers, together with Pleistocene climatic fluctuations and shifts in vegetation belts, may have acted synergistically to promote diversification, as documented in other vertebrate groups in America (Magalhães et al., 2022; Krabbe et al., 2020).

Both regions where the new species are found represent high priority zones for conservation and connectivity in the Ecuadorian Andes, due to their significant levels of endemism and biodiversity. This includes Mira Mataje Basin for *Niceforonia dracula* sp. nov. (Yánez-Muñoz et al., 2024), and the upper Pastaza with Llanganates-Sangay for *N. quilloturo* sp. nov. (Ríos-Alvear et al., 2024). However, threats such as mining and deforestation are rapidly increasing, putting the populations of the new species and their habitats at risk.

Declaration of new protected areas is imperative to ensure connectivity between National Parks and the different kinds of reserves. This will ensure the preservation of genetic diversity and endemism hotspots of the genus *Niceforonia* in Ecuador.

Our work has made significant steps in the understanding of this group of terrestrial anurans, but certainly much work remains. Resolving taxonomic issues for the candidate new taxa will require morphological revisions, with special attention in the shape and structure in the tip of fingers, toes and vomerine odontophores. *Niceforonia dracula* is the only species with large expanded toes with circunmarginal grooves, 9–10 transverse vomerine odontophores, while its sister species, *N. elassodiscus* bears 5–8 transverse vomerine odontophores and rounded tip of digits and toes (Lynch, 1973). *Niceforonia quilloturo* sp. nov., on its turn present oblique or arched vomerine odontophores and its sister species *N. peraccai* bears 0–7 transverse vomerine odontophores and tip of digits and toes with more acute terminations (Lynch, 1975).

Future studies of the genus should aim for new samples from diverse localities and generate osteological computed tomography (CT) scans for all taxa. This approach will help describe differences in osteology and specific variations among the clades, particularly within the taxonomic complex of *Niceforonia nigrovittata*, which is a highly cryptic group. The combination of genetic, morphological, ecological, and osteological analysis has successfully addressed similar cases of cryptic diversity (Franco-Mena et al., 2024; Ortega, Brito & Ron, 2022), providing us with more detailed characters for a better understanding of the genus in the future.

## Conclusions

Our work updates the knowledge of the *Niceforonia* genus in Ecuador, revealing two new species remarkably different from its relatives based on genetic, morphology and geographic distribution evidence.

Phylogenetic relationships of the genus show multiple candidate taxa undermixed with the main clades, and a future big challenge is the resolution of the *Niceforonia nigrovittata* complex.

Until new sequences of Colombian species become available, a wide diversification is evident eastern to the Andes in Ecuador, reflecting a center of radiation and endemism, parapatric distributions of the clades are consistent with mountain ranges barriers and hydrographic configuration of the eastern slopes of the Andes.

## Additional Information and Declarations

### Field Study Permissions

The following information was supplied relating to field study approvals (*i.e*., approving body and any reference numbers):

Collections was aproved by Environmental Ministry of Ecuador thoruhg collection permits MAE DNB CM 2019-0120, MAATE ARSFC 2023 3346, MAATE-ARSFC

2024-0847.

### Data Availability

The following information was supplied regarding data availability:

The sequences are available at NCBI:

- QCAZA30792: PX629809

- QCAZA42398: PX629810

- QCAZA59404: PX629811

- QCAZA78324: PX629812

- QCAZA78325: PX629813

- QCAZA78654: PX629814

- QCAZA78657: PX629815

- QCAZA78662: PX629816

- DHMECN14417: PX629817

- DHMECN14418: PX629818

- DHMECN17174: PX629819

- DHMECN17175: PX629820

- DHMECN17330: PX629821

- DHMECN18852: PX625814

- DHMECN18853: PX625815

- DHMECN19273: PX625816

- DHMECN19274: PX625817

- DHMECN19347: PX625818

- DHMECN19406: PX625819

- DHMECN19881: PX625820

- DHMECN19882: PX625821

- DHMECN19883: PX625822

- DHMECN19884: PX625823

- DHMECN19885: PX625824

- DHMECN19886: PX625825

- DHMECN19995: PX625826

- DHMECN20003: PX625827

- QCAZA25789: PX625828

- QCAZA41046: PX625829

- QCAZA41054: PX625830

- QCAZA41068: PX625831

- QCAZA41146: PX625832

- QCAZA41299: PX625833

- QCAZA52497: PX625834

- QCAZA57536: PX625835

- QCAZA66306: PX625836

- QCAZA68992: PX625837

- DHMECN19071: PX596790

- DHMECN19072: PX596791

- DHMECN19073: PX596792

- DHMECN19075: PX596793

- DHMECN19269: PX596794

- DHMECN19407: PX596795

- DHMECN19408: PX596796

- DHMECN19409: PX596797

### New Species Registration

The following information was supplied regarding the registration of a newly described species:

- Publication LSID: urn:lsid:zoobank.org:pub:1E0F3CA8-699C-4618-B137-53017A2560C2

- Niceforonia dracula (dracula andean frog) LSID: urn:lsid:zoobank.org:act:17F177F5-7A76-4C49-BA63-503D42D2D24A

- Niceforonia quilloturo (muddy narrow toed frog) LSID: urn:lsid:zoobank.org:act:21F4FE0C-4D5A-460B-B9C6-18BD639E9DE0.
